# The presence of human polyomavirus JC (JCPyV) in pediatric brain tumors: a plausible trigger in Wnt/β-catenin pathway

**DOI:** 10.1007/s13365-025-01274-7

**Published:** 2025-09-17

**Authors:** Sara Passerini, Sara Messina, Marta De Angelis, Lucia Nencioni, Francesca Gianno, Manila Antonelli, Valeria Pietropaolo

**Affiliations:** 1 Department of Public Health and Infectious Diseases, Sapienza University of Rome, Rome, 00185 Italy; 2 Department of Public Health and Infectious Diseases, Sapienza University, Laboratory Affiliated to Istituto Pasteur Italia-Fondazione Cenci Bolognetti, Rome, Italy; 3 Laboratory of Virology, Department of Molecular Medicine, Sapienza University, Rome, Italy; 4 Department of Radiological, Oncological and Anatomo‑Pathological Sciences, “Sapienza” University of Rome, Rome, Italy; 5 IRCCS Neuromed, Pozzilli, Italy

**Keywords:** JC polyomavirus, Brain tumors, Wnt/ß-catenin pathway, Oncogenesis

## Abstract

JC polyomavirus (JCPyV) is associated with progressive multifocal leukoencephalopathy (PML), but its plausible role in brain cancers is also disputed. One candidate to mediate cell transformation is the Large T antigen (LTAg), which has the capability to bind the Wnt pathway protein β-catenin, thus deregulating the cell cycle. In the current study, we investigated the presence and molecular state of JCPyV in pediatric brain tumors and the effects of virus-positivity on the Wnt pathway. JCPyV DNA was found in 31/101 (30.7%) brain tumors with a viral load of 3.2 copies/cell. The amplified NCCR revealed an archetype sequence, and VP1 reported a high degree of homology with the reference strain. The *LTAg* gene was reported in all JCPyV-positive tumors. Interestingly, among them, 5 tissues did not express *VP1* and viral miRNAs, supporting a hampering of late region transcription. Over-expression of *β-catenin*, *c-myc* and *cyclin D1* was observed in JCPyV-positive tissues compared to negative ones, suggesting that the virus may exploit this signaling pathway, potentially contributing to brain carcinogenesis. The current study adds further evidence of JCPyV prevalence in human brain tumors and reports alterations of the Wnt pathway, laying the basis for further investigation on JCPyV-mediated oncogenesis in the brain.

## Introduction

Central Nervous System (CNS) tumors are a wide heterogeneous type of tumors originating from the brain or spinal cord. According to their features, CNS tumors are classified using histological findings and molecular biomarkers into different types and subtypes and different grades (I-IV) by the WHO Classification (Louis et al. 2021). The etiology of these tumors is still not fully defined. Several risk factors have been proposed, including genetic, environmental and lifestyle factors (Wrensch et al. 2002; Bondy et al. 2008; Ostrom et al. 2019). In addition, since a wide range of viral infections cross the blood-brain-barrier, a viral etiology has also been suspected (Egan et al. 2021). Several viruses, including Epstein-Barr virus (EBV), Human Papillomavirus (HPV) and Polyomaviruses (PyVs) have been isolated in adults and pediatric gliomas, supporting their role in brain cancers (Egan et al. 2021; Okamoto et al. 2005; Vidone et al. 2014; Limam et al. 2019b). Human Polyomaviruses (HPyVs) are small, non-enveloped DNA viruses widely distributed among population (DeCaprio And Garcea 2013). Previous studies demonstrated that many members of this family possess transforming activity, such as Merkel Cell Polyomavirus (MCPyV), well known to be the etiological agent of Merkel Cell Carcinoma (MCC), a rare but aggressive carcinoma of the skin (Prado et al. 2018; DeCaprio 2021). Moreover, BK virus (BKPyV) and JC virus (JCPyV), which are phylogenetically related, have been identified in glial tumors, supporting their possible involvement in the pathogenesis of human brain cancers (Huang et al. 1999; Kouhata et al. 2001; Del Valle et al. 2001; White et al. 2005). JCPyV was isolated, for the first time, in 1971 from the brain tissue of a patient with Hodgkin’s lymphoma (Padgett et al. 1971) and represents the etiologic agent of the progressive multifocal leukoencephalopathy (PML), a CNS demyelinating disease (Ferenczy et al. 2012; Pietropaolo et al. 2018).

The JCPyV genome contains three functional domains: the early and the late coding regions and the Non-Coding Control Region (NCCR), interposed between them (Frisque et al. 1984; Khalili 2001; Moens et al. 2017; Ciotti et al. 2019). The early region encodes for the Large Tumor-Antigen (LTAg), small T antigen (stAg) and T’ proteins, which are involved in the regulation of the virus cycle and cell transformation (Frisque et al. 1984; Ciotti et al. 2019; Tan And Koralnik 2010). The late region produces the capsid protein VP1, VP2, VP3, the accessory Agnoprotein and two mature microRNAs (miRNAs), JC-miR-3p and JC-miR-5p, able to control viral replication by downregulating LTAg expression (Trowbridge And Frisque 1995; Seo et al. 2008). The NCCR, encompassing the origin of replication (ORI) and transcription control sequences, is a hypervariable region that contributes to neurotropism and neurovirulent properties of JCPyV (Pietropaolo et al. 2018; Moens et al. 2020). The archetype NCCR structure is the transmissible form of the virus (Yogo et al. 1990), whereas the rearranged forms are frequently disease-associated (Frisque et al. 1984; Moens et al. 2020). Either Mad-1 or Mad-4 NCCR variants have been identified in JCPyV-positive human brain tumors; interestingly, the archetype structure has also been observed (Passerini et al. 2023).

The JCPyV principal actor, leading to cell transformation and tumor development, is the LTAg, an early protein essential for viral DNA replication (White et al. 2005)that interferes with a series of cell regulatory proteins, responsible for driving cells into S phase (Moens et al. 2022; Del Valle and Piña-Oviedo 2019). LTAg N-terminal region, contains the LXCXE and DnaJ domains, necessary for binding and inactivating the Retinoblastoma (Rb) family members, whereas the C-terminal region contains the p53-binding domain (Sharma And Kumar 1991; Zheng et al. 2022). Specifically, Rb binding promotes cell cycle progression, whereas the LTAg-p53 complex leads to the inhibition of apoptosis (Moens et al. 2022). It was demonstrated that JCPyV LTAg can also interact with β-catenin and co-localize in the nuclei, where it enhances transcription of β-catenin downstream targets, such as c-myc and cyclin D1 (Gan and Khalili 2004; Ripple et al. 2014). The exact mechanism by which LTAg disrupts the β-catenin signaling pathway remains unclear. However, it is hypothesized that LTAg may stabilize β-catenin by preventing its degradation through the proteasome (Gan et al. 2001; Moens And Macdonald 2019). Another possibility is that LTAg promotes the nuclear translocation of β-catenin, as supported by the observation that β-catenin is more frequently localized in the nucleus of JCPyV LTAg-positive cancers compared to LTAg-negative tumors (Ripple et al. 2014). Therefore, it is clear that JCPyV LTAg’s ability to interfere with β-catenin may contribute to the oncogenic potential of this virus.

To date, there are both experimental and clinical evidence for a role of JCPyV in cancer (Del Valle and Khalili 2021). JCPyV is able to transform cells of glial origin, giving them the typical phenotype associated with transformation (Del Valle et al. 2008; An et al. 2012). Moreover, studies in animal models showed the highly oncogenic potential of this virus (Khalili 2001). Several investigators have also reported viral sequences and/or protein expression in a wide variety of glial tumors such as glioblastoma, medulloblastoma, pleomorphic xanthoastrocytoma, oligodendroglioma, ependymoma and all subtypes of astrocytoma (Passerini et al. 2023; Krynska et al. 1999; Del Valle et al. 2002, 2002; Delbue et al. 2005; Dehghan Manshadi et al. 2018). Despite the increasing evidence of an association between JCPyV infection and tumor development, different studies failed to detect viral genome and protein expression in CNS tumors (Muñoz-Mármol et al. 2006). Moreover, due to its ubiquitous nature, JCPyV was also detected in the brain of healthy subjects (Perez-Liz et al. 2008; Delbue et al. 2008). Consequently, the causative role of JCPyV in brain cancers remains to be defined.

Given this background, in this study, we investigated the presence and molecular state of JCPyV, and the expression of *β-catenin*, *c-myc* and *cyclin D1* in a series of pediatric brain tumors to investigate whether JCPyV is involved in tumorigenesis as an underlying cancerogenic or co-cancerogenic factor in the complex processes of glial tumour induction and development.

## Materials and methods

### Clinical samples

Formalin-fixed paraffin-embedded (FFPE) tissue sections from glioma biopsies were collected from 101 pediatric patients (57 males and 44 females, mean age 12.8 ± 11.4) as part of the Italian National Program of Centralization of Pediatric Brain Tumor. Specimens were chosen based on the accessibility of complete clinical data and the availability of FFPE tissues for analysis. To ensure adequate tumor content, hematoxylin and eosin (H & E) slides were reviewed after the initial cut of each FFPE block for DNA extraction. This research study was conducted retrospectively from data obtained for clinical purposes. Moreover, the study was performed according to local Ethical and Institutional Review Board approval (Protocol Number: 1337/20), and informed consent was obtained in accordance with the Declaration of Helsinki. The demographic and clinical characteristics of patients are listed in Table 1.

**Table 1:** Demographic and clinical characteristics of patients

Features	Population	
Patients	101	
Sex, *n* (%)	M	F
	57 (56.4%)	44 (43.6%)
Mean age, years (SD)	12.8 (± 11.4)	
Tumor type, *n*		
Adamantinomatous craniopharyngioma (gr. I)	1	
Anaplastic astrocytoma (gr. II)	1	
Anaplastic astrocytoma (gr. III)	13	
Pilocytic astrocytomas (gr. I)	13	
Pilocytic astrocytomas (gr. III)	1	
Anaplastic medulloblastoma (gr. IV)	2	
Anaplastic pleomorphic xanthoastrocytoma (PXA) (gr. III)	1	
Central neurocytoma (gr.II)	1	
Medulloblastoma (gr. IV)	4	
Diffuse astrocytoma (gr. II)	7	
Ependymoma (gr. II)	6	
Anaplastic ependymoma (gr. III)	3	
Ganglioglioma (gr. I)	1	
Anaplastic medulloblastoma (gr. IV)	2	
Glioblastoma (gr. IV)	27	
Gliosarcoma (gr. IV)	1	
Anaplastic Xanthoastrocytoma (gr. III)	1	
Pleomorphic Xanthoastrocytoma (gr. II)	3	
Emangioblastoma (gr. I)	1	
Neuroblastoma	1	
High-grade glioma	8	
Low-grade glioma	3	

### DNA extraction

After deparaffinization with Xylene, total DNA extraction was performed using Quick-DNA FFPE Miniprep (Zymo Research, Irvine, CA) according to the manufacturer’s instructions. The extracted nucleic acid was eluted in a final volume of 50 µl, and DNA was evaluated for its PCR suitability by amplifying the *β-globin *gene sequences (Saiki et al. 1985).

### Detection of JCPyV DNA by real time polymerase chain reaction (qPCR)

The presence and quantity of viral DNA in FFPE sections were evaluated by quantitative polymerase chain reaction (qPCR), able to detect a 54 bp amplicon in JCPyV LTAg region (Delbue et al. 2008; Arthur et al. 1989). The standard curve was obtained from serial dilutions (range: 10^5^–10^2^ copies/ml) of a plasmid containing the entire JCPyV genome. Each sample was analyzed in triplicate, and JCPyV DNA loads (given as the mean of at least three positive reactions) were expressed as copies/cell. Specifically, a standard curve of *β-globin* was used for cell quantification, and JCPyV copies were normalized by calculating the ratio of viral copy numbers to half of *β-globin* gene copy numbers. Precautions to prevent contamination were followed, and a negative control was included in each qPCR session.

### Detection and sequencing of other region of the JCPyV genome

JCPyV-positive samples were further analyzed using PCR for NCCR and VP1 regions’ amplification (Flægstad et al. 1991; Markowitz et al. 1993; Agostini et al. 1997; Prezioso et al. 2018). The PCR products were analysed on a 2% agarose gel and were visualized using SafeView reagent (Applied Biological Materials, Vancouver, BC, Canada) under UV light. In addition, in order to assess NCCR and VP1 variations, the amplified products were purified using the miPCR purification kit (Metabion, Plannegg, Germany) and sequenced in a dedicated facility (Bio-Fab Research, Rome, Italy). Obtained sequences were compared to the reference strain (GenBank: AB038249). Sequence alignment was performed using ClustalW2 on the European Molecular Biology Laboratory–European Bioinformatics Institute (EMBL–EBI) website using default parameters (ClustalW2 2025). Moreover, JCPyV genotypes were classified based on single-nucleotide polymorphisms (SNPs) found within the amplified VP1 region (Pagani et al. 2003).

### RNA extraction and analysis

Following deparaffinization with Xylene, total RNA, including miRNA, was extracted from the FFPE tissue sections, using the Total RNA purification kit (Norgen, Thorold, ON, Canada). RNA quality and quantity were assessed using A230/A260 ratios. The RNA was then reverse-transcribed, and an aliquot of the reverse transcription reaction mixture was used for PCR amplification to examine JCPyV *LTAg* and *VP1 *genes’ expression (Passerini et al. 2023).

### Viral MiRNAs detection

Viral miRNAs detection was performed using the specific Applied Biosystems™ TaqMan™ MicroRNA Assays (Thermo Fisher Scientific, Waltham, MA, USA). Specifically, the pre-designed TaqMan microRNA assay for jcv-miR-J1-5p (ID 007464) was employed. Moreover, the human miRNA RNU6B (ID001093) was included as an endogenous control for extraction and reverse-transcription efficacy.

### Analysis of β-catenin, c-myc and cyclin D1 genes

RT-qPCR was performed for *β-catenin*, *c-myc* and *cyclin D1 *(Sareddy et al. 2009). The housekeeping *GAPDH* gene was used as an internal quantitative control to normalize the obtained mRNA levels, and the relative expression of the genes was expressed as 2^−ΔCt^.

### Statistical analysis

For statistical analysis, data were expressed as the mean ± standard deviation or as median and interquartile range (IQR). Viral loads and gene expression were tested using the Shapiro–Wilk test to assess normal distribution. Viral loads were compared by Mann-Whitney U for unmatched data, whereas mean values of Wnt target genes were compared by Student’s *t*-test. *p* < 0.05 was considered statistically significant.

## Results

### Detection of JCPyV DNA in brain tumor tissues

JCPyV DNA was found in 31/101 (30.7%) tumor biopsies with a median viral load of 3.2 (IQR: 0.9–14) copies/cell. JCPyV was isolated at a higher rate (*n* = 21/31, 67.7%) in high-grade tumors. However, when comparing viral loads in high and low-grade tumors, no significant differences were observed (*p* > 0.05) (Table 2).

**Table 2:** JCPyV viral load and molecular state in JCPyV-positive tumors

Case *n*°	Diagnosis	Viral load (copies/cell)	Viral Sequences		Viral Transcripts		
			NCCR	VP1	LT	VP1	miR-5p
5	Anaplastic Xanthoastrocytoma (gr. III)	247	archetype	1 A	P	N	N
6	Pilocytic astrocytoma (gr. I)	1.09	archetype	1 A	P	P	P
7	Anaplastic ependimoma (gr. III)	11	archetype	1B	P	N	N
8	Ganglioglioma (gr. I)	41.7	archetype	1 A	P	N	N
9	Central neurocytoma (gr. II)	19.3	archetype	1B	P	N	N
10	Glioblastoma (gr. IV)	0.7	archetype	1 A	P	P	P
11	Anaplastic astrocytoma (gr. III)	3.2	archetype	1B	P	P	P
12	Glioblastoma (gr. IV)	1.7	archetype	1 A	P	P	P
13	Glioblastoma (gr. IV)	0.3	archetype	1B	P	P	P
14	Glioblastoma (gr. IV)	3.2	archetype	1 A	P	P	P
15	High-grade glioma	0.8	archetype	1 A	P	P	P
16	Gliosarcoma (gr. IV)	0.9	archetype	1 A	P	P	P
20	Diffuse astrocytoma (gr. II)	0.7	archetype	1B	P	P	P
21	Glioblastoma (gr. IV)	2.35	archetype	1B	P	P	P
22	Anaplastic astrocytoma (gr. III)	7.97	archetype	1 A	P	P	P
23	Diffuse astrocytoma (gr. II)	53.4	archetype	1B	P	N	N
28	Diffuse astrocytoma (gr. II)	3.4	archetype	1 A	P	P	P
29	High-grade glioma	0.9	archetype	1B	P	P	P
57	High-grade glioma	3.1	archetype	1B	P	P	P
59	Glioblastoma (gr. IV)	2.1	archetype	1 A	P	P	P
60	Glioblastoma (gr. IV)	14	archetype	1 A	P	P	P
62	Glioblastoma (gr. IV)	9.6	archetype	1B	P	P	P
63	Glioblastoma (gr. IV)	3.3	archetype	1B	P	P	P
74	Low-grade glioma	16.6	archetype	1 A	P	P	P
82	Glioblastoma (gr. IV)	59.9	archetype	1B	P	P	P
85	Pilocytic astrocytoma (gr. III)	1.84	archetype	1 A	P	P	P
93	Glioblastoma (gr. IV)	93.3	archetype	1B	P	P	P
95	Ependimoma (gr. II)	0.65	archetype	1 A	P	P	P
96	Pilocytic astrocytoma (gr. I)	4.6	archetype	1 A	P	P	P
98	Pilocytic astrocytoma (gr. I)	0.6	archetype	1B	P	P	P
99	High-grade glioma	3.25	archetype	1 A	P	P	P

### Analysis of JCPyV NCCR and VP1 structure

The amplified JCPyV NCCRs were aligned to the reference sequence, revealing an archetype structure in all samples tested positive for JCPyV DNA detection. VP1 also showed a high degree of homology with the reference strain. Specifically, sequence analysis identified the European genotype 1 A in 17/31(54.8%) and the 1B in 14/31 (45.2%) virus-positive samples (Table 2).

### Expression of transcripts from LTAg and VP1 genes

JCPyV-positive tumors were further subjected to transcript analysis of early and late genes by RT-PCR. All samples showed the expression of *LTAg*, whereas *VP1* was detected in 26/31 (83.9%) JCPyV-positive tissues (Table 2).

### Viral MiRNAs detection

Among JCPyV DNA-positive samples, jcv-miR-J1-5p was found in 26/31 (83.9%) samples. More specifically, viral miRNAs were detected in the same 26 tissues tested positive for *VP1* gene expression (Table 2).

### Relative expression of Wnt target genes

**Fig. 1:**
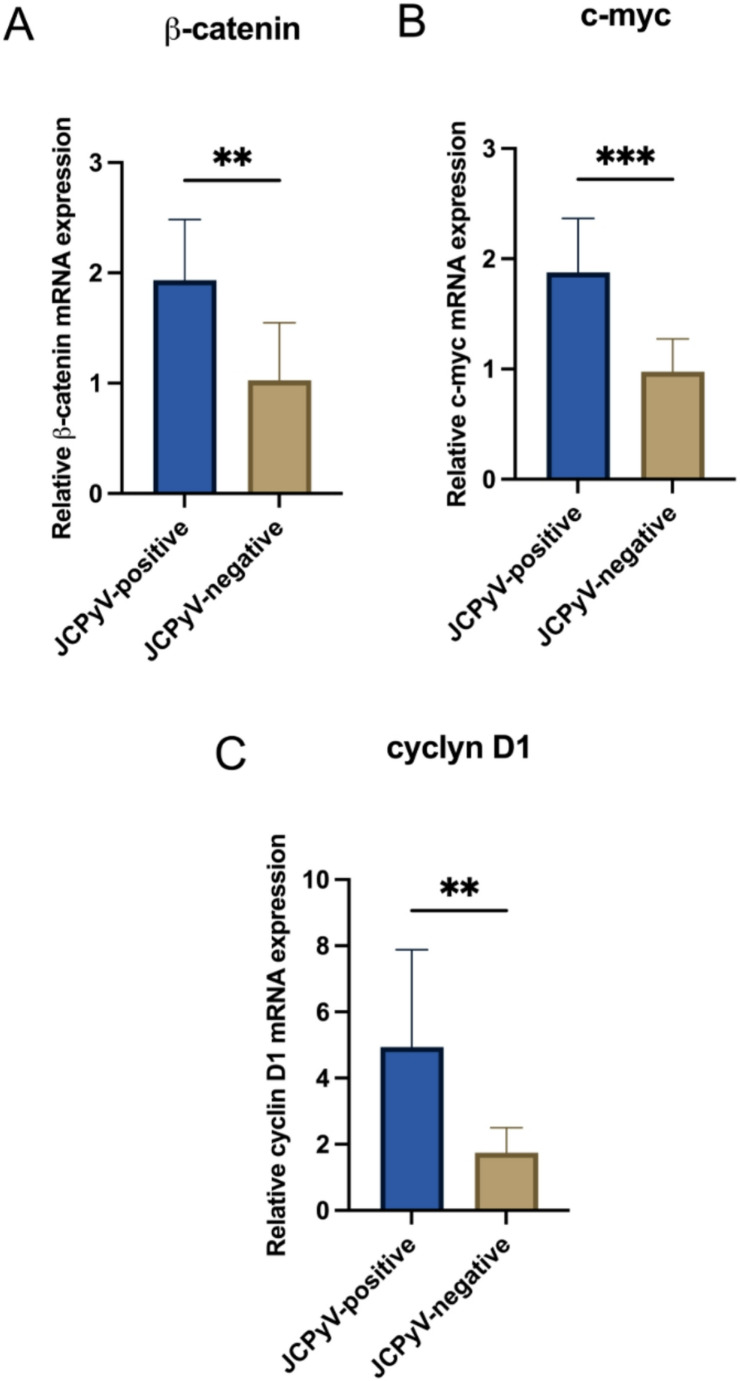
Relative expression of *β-catenin*, *c-myc* and *cyclin D1* mRNAs in JCPyV-positive and JCPyV-negative brain tumors. Mean values of Wnt target genes (*β-catenin*, Panel A; *c-myc*, Panel B; *cyclin D1*, Panel C) in JCPyV-positive and negative groups were compared by independent T-test. *p < 0.05; **p < 0.01; ***p ≤ 0.001

When analyzing *β-catenin*, *c-myc* and *cyclin D1* genes’ expression, a significant over-expression was observed in JCPyV-positive tissues compared to virus-negative ones (*p* < 0.05) (Fig. 1).

## Discussion

CNS tumors are highly heterogeneous neoplasms with poor understood etiology, with both genetic and environmental factors contributing to their pathogenesis (Ostrom et al. 2019). Among risk factors, oncogenic viruses have emerged as plausibly involved (Egan et al. 2021; Okamoto et al. 2005; Vidone et al. 2014; Limam et al. 2019b). JCPyV is a human neurotropic virus, well recognized as the causative agent of PML, but its contribution to the development of human brain tumors is also disputed (Del Valle et al. 2008; Ahye et al. 2020). Viral DNA, transcripts and proteins have been found in brain cancers, supporting a plausible correlation between JCPyV infection and tumor development (Passerini et al. 2023; Krynska et al. 1999; Del Valle et al. 2002, 2002; Delbue et al. 2005; Dehghan Manshadi et al. 2018). However, whether JCPyV conclusively plays a role in CNS carcinogenesis is still a topic of debate.

In the current study, CNS tumors tissues from pediatric patients were examined for JCPyV prevalence and molecular state. The distribution of different tumor types reflects the epidemiological state of the Italian population, in which glioblastoma represents the most frequent type of brain tumor (Frosina et al. 2024). Viral DNA was isolated from about 31% of the analyzed samples, supporting the prevalence of the virus in CNS tumors. A higher positivity rate of JCPyV was observed in high-grade tumors, likely due to the overrepresentation of this tumor type among the analyzed specimens. Additionally, when comparing viral loads between high-grade and low-grade cancers, no significant differences were observed.

Moreover, in order to explore the plausible correlation between virus-positivity and brain malignancies, viral sequences were examined, and transcripts analysis was performed. Mutations in the NCCR sequence have been previously associated with brain malignancies; specifically, oncogenic properties have been recognized for Mad-4 rearrangements (Delbue et al. 2005; Gordon et al. 2000). Interestingly, archetype NCCR structures were identified from JCPyV-positive tumors, suggesting that mutations within NCCR architecture may not be directly associated with virus-mediated oncogenesis (Passerini et al. 2023). Moreover, molecular analysis of the VP1 region revealed highly conserved sequences; specifically, genotype 1 was isolated from JCPyV-positive tumors, confirming it as predominant overall Italian population (Muñoz-Mármol et al. 2006; Pagani et al. 2003). Transcripts analysis revealed the expression of both early and late genes in the majority of the cases. In 5 virus-positive tumors, instead, only *LTAg* is expressed with no detection of *VP1 *transcripts. A similar pattern of viral gene expression is observed in MCPyV-associated MCC, where hampering of viral replication is considered a hallmark for MCPyV-related oncogenesis (Zur Hausen 2008; Passerini et al. 2023). Based on these observations, it is rational to propose that, as for MCPyV, loss of viral replication, supported by the absence of *VP1 *detection at the RNA level, may contribute to JCPyV-mediated brain carcinogenesis (Del Valle et al. 2008). In addition to early and late genes transcription, viral miRNAs expression was tested. Since viral miRNAs have been proposed as plausible biomarkers for JCPyV infection, reducing LTAg expression during viral persistence (Lagatie et al. 2013; Prezioso et al. 2022), we may speculate that the detection of miR-5p among the majority of virus-positive tumors (*n* = 26, 83.9%), alongside the detection of an archetype NCCR and both *LTAg* and *VP1 *genes contributes to a persistent infection rather than viral reactivation. Despite JCPyV miR-5p is known to have a key role in silencing LTAg expression (Lagatie et al. 2013; Martelli et al. 2018), its concomitant detection with both early and late transcripts in JCPyV-positive tumors, may reflect a fine-tuning of viral persistence in which miRNA-mediated suppression modulates but does not completely silence viral transcription. This suggests a complex interplay between viral gene expression and persistence, plausibly influenced by cellular microenvironment or infection stage. Based on these results, it could be hypothesized that the persistent presence of JCPyV in brain tissues may lead to the occurrence of mutations and chromosomal instability as an earlier phase for tumorigenesis (Okamoto et al. 2005). Notably, viral miRNAs were undetectable in the 5 brain tissues negative for *VP1*, supporting a hampering of late region transcription.

Although the biological significance of JCPyV infection in the genesis of CNS cancer remains unclear, the detection of viral DNA and RNA in tumor tissues corroborates the plausible involvement of JCPyV in brain carcinogenesis. However, the isolation of the virus, while indicative of possible involvement of the virus in brain tumors, is not sufficient to define the actual contribution of the virus to neoplasm progression.

Therefore, once assessed JCPyV prevalence and molecular state in CNS tumors, another aim of the study was to evaluate the effect of JCPyV infection on Wnt/*β-catenin *pathway components, to further understand the contribution of the virus in the context of brain cancer. Dysfunction of the canonical Wnt pathway is recognized as a trigger factor for carcinogenesis of CNS tumors (Sareddy et al. 2009; Suh And Choi 2024), and JCPyV has been found as capable to modulate this cellular signaling (Gan and Khalili 2004; Ripple et al. 2014; Gan et al. 2001). Earlier studies indicated that JCPyV LTAg disrupts Wnt signaling in medulloblastoma; specifically, enhanced *β-catenin *transcription has been observed following LTAg DNA transfection (Gan et al. 2001). Moreover, the nuclear translocation of β-catenin has been reported in JCPyV-positive tumors (Ripple et al. 2014; Enam et al. 2002). Our findings showed an over-expression of *β-catenin* mRNA in JCPyV-positive tissues. As the core component of the Wnt canonical signaling pathway, *β-catenin *plays a crucial role in cell proliferation, cell-cell adhesion, tumor cell migration and invasion (Liu et al. 2022). Therefore, its over-expression in virus-positive tumors support that JCPyV may induce the transcriptional activation of *β-catenin* thus contributing to perturb Wnt signaling pathway. To further examine the effect of JCPyV-positivity on Wnt pathway, the relative levels of *β-catenin* target genes, *c-myc* and *cyclin D1 *were assessed. Similarly, an enforced expression of these two proto-oncogenes was observed in virus-positive tissues, endorsing that JCPyV contributes to their transcriptional activation (Ripple et al. 2014; Gan et al. 2001). In the context of JCPyV infection, the high levels of *c-myc* and *cyclin D1* may be a result of virus-driven activation of Wnt/β-catenin pathway, thus supporting the role of the virus in exploiting cell machinery for inducing tumorigenesis. However, as mRNA analysis, despite highly sensitive, does not directly confirm the activation of Wnt signaling pathway by JCPyV, future studies exploring the interactions between JCPyV LTAg and β-catenin in CNS tumors will strongly contribute to clarify JCPyV relevance in brain carcinogenesis. Moreover, the absence of a control group represents a limitation of the study that prevent a comprehensive assessment of the association between JCPyV infection and brain carcinogenesis. However, collecting tissues from pediatric patients without brain tumors poses major challenges due to ethical concerns.

## Conclusions

In conclusion, the prevalence of JCPyV in tumor tissues and, more importantly, the alteration of Wnt pathway components in virus-positive cancers reinforces the plausible role, perhaps as a co-cancerogenic factor, in the development and/or progression of CNS tumors. Based on the obtained results, further studies in vitro are currently in progress to explore the mechanistic role of JCPyV infection on Wnt pathway components in the context of brain tumors.

## Data Availability

The datasets containing all data analysed, supporting the results of this study, will be made available by the authors, without undue reservation.
