# Correction: The presence of human polyomavirus JC (JCPyV) in pediatric brain tumors: a plausible trigger in Wnt/β-catenin pathway

**DOI:** 10.1007/s13365-025-01284-5

**Published:** 2025-09-26

**Authors:** Sara Passerini, Sara Messina, Marta De Angelis, Lucia Nencioni, Francesca Gianno, Manila Antonelli, Valeria Pietropaolo

**Affiliations:** 1 Department of Public Health and Infectious Diseases, Sapienza University of Rome, Rome, 00185 Italy; 2 Department of Public Health and Infectious Diseases, Sapienza University, Laboratory Affiliated to Istituto Pasteur Italia-Fondazione Cenci Bolognetti, Rome, Italy; 3 Laboratory of Virology, Department of Molecular Medicine, Sapienza University, Rome, Italy; 4 Department of Radiological, Oncological and Anatomo Pathological Sciences, “Sapienza” University of Rome, Rome, Italy; 5 IRCCS Neuromed, Pozzilli, Italy

**Correction:** **Journal of NeuroVirology**

**https://doi.org/10.1007/s13365-025-01274-7**

Author Manila Antonelli has two affiliations, affiliations 4 and 5: Department of Radiological, Oncological and Anatomo Pathological Sciences, “Sapienza” University of Rome, Rome, Italy and IRCCS Neuromed, Pozzilli, Italy.

